# Cancer Cell Lines Are Useful Model Systems for Medical Research

**DOI:** 10.3390/cancers11081098

**Published:** 2019-08-01

**Authors:** Peppino Mirabelli, Luigi Coppola, Marco Salvatore

**Affiliations:** IRCCS SDN, 80143 Naples, Italy

**Keywords:** cell lines, solid cancer, leukemia, medical research, biological samples

## Abstract

Cell lines are in vitro model systems that are widely used in different fields of medical research, especially basic cancer research and drug discovery. Their usefulness is primarily linked to their ability to provide an indefinite source of biological material for experimental purposes. Under the right conditions and with appropriate controls, authenticated cancer cell lines retain most of the genetic properties of the cancer of origin. During the last few years, comparing genomic data of most cancer cell lines has corroborated this statement and those that were observed studying the tumoral tissue equivalents included in the The Cancer Genome Atlas (TCGA) database. We are at the disposal of comprehensive open access cell line datasets describing their molecular and cellular alterations at an unprecedented level of accuracy. This aspect, in association with the possibility of setting up accurate culture conditions that mimic the in vivo microenvironment (e.g., three-dimensional (3D) coculture), has strengthened the importance of cancer cell lines for continuing to sustain medical research fields. However, it is important to consider that the appropriate use of cell lines needs to follow established guidelines for guaranteed data reproducibility and quality, and to prevent the occurrence of detrimental events (i.e., those that are linked to cross-contamination and mycoplasma contamination).

## 1. Introduction

Cancer cell lines are valuable in vitro model systems that are widely used in cancer research and drug discovery [1]. Their use is primarily linked to their peculiar capability to provide an indefinite source of biological material for experimental purposes [2].

The establishment of a new cell line is a very complex process that is still not well understood. The success rate for the establishment is low and unpredictable for any specimen of origin [3]. This statement could seem paradoxical when considering that the stabilization of a cell line starts with a sample of tumors able to grow vigorously in vivo, escaping all cellular mechanisms that are involved in the control of the cell cycle and cell death by apoptosis [4,5,6,7]. However, many causes of this difficulty and serendipity for the establishment of a new cell line can be understood by taking into consideration the extreme differences (such as growth factor dependence, the percentage of oxygen, interaction with the stroma and immune cells, etc.) that exist between the in vivo and in vitro microenvironments [3]. This issue is witnessed by the impossibility of establishing, for example, a continuous cell line from chronic myeloid leukemia in the chronic phase. This hematological disorder is characterized by a very high rate of proliferation of leukemic cells in vivo, but the same leukemic cells die after a few weeks in vitro [3]. Furthermore, regarding the success of continuous growth in vitro, the procedure for the establishment of a new cell line is, in any case, difficult and time consuming, requiring even more than one or two years [8,9,10]. Nevertheless, since each cell line is derived from the disease from which the patient is suffering from, its offers the opportunity for disclosing pathological features that were otherwise unidentified by conventional clinical diagnostic settings [11] and to perform experiments that are not possible to be performed in vivo. The processes of stabilizing and characterizing a new cell line should be performed in agreement with published guidelines. In particular, in 1999, Drexler and Matsuo published the “Guidelines for the characterization and publication of human malignant hematopoietic cell lines” and stressed the importance of confirming the immortality, authenticity, and tissue or cell type of origin for each newly established cell line [8]. These guidelines are still valid and they are included in the updated United Kingdom Coordinating Committee on Cancer Research (UKCCCR) guidelines for the use of cancer cell lines in biomedical research published by Geraghty et al. in 2014 [10]. Indeed, a detailed characterization, the immortality of the culture, a proof of neoplasticity, authentication of the true origin of the cells, scientific significance and availability of the cell line for other investigators are of paramount importance when publishing a new cell line. In this way, under the right conditions and with appropriate controls, properly authenticated cancer cell lines retain most of the properties of the cancer of origin [1] and they become helpful model systems for the progress of medical research [12,13].

Recent findings that were obtained by the characterization of hundreds of cell lines with omics technologies (i.e., genomics, transcriptomics, and proteomics) reinforced the concept of cell line usefulness in medical research. Indeed, for the majority of the existing cancer cell lines, these data have been published and made available through online datasets, which made it possible to explore detailed molecular and cellular alterations, such as mutations [14], copy number variations [15], and gene [16] and protein [17] expression profiles, featuring each cell line. In this way, the process for selecting the most appropriate model systems for experimental purposes has been significantly enhanced [18]. In this context, we must not forget that the right use of continuous cell lines, following appropriate guidelines [10], is mandatory. Indeed, as in the past, the quality and reproducibility of research data could be irreparably compromised if cross-contaminated or misidentified cell lines [19] were used and/or when cultures were contaminated with mycoplasma [20].

## 2. The Historical Progress of Cell Lines: Important Breakthroughs for Medical Research

The history of cancer research and the establishment of continuous cell lines are closely related [1]. Different investigators have begun to understand the complex mechanisms that transform a normal cell into a cancer cell and subsequent tumor development due to the availability of these precious models. From a historical point of view, tissue culture techniques were established at the beginning of the 20th century when the first work describing the culture of living tissues was reported by Harrison at the Anatomical Department of Johns Hopkins University in 1907 [21]. In a series of in vitro experiments, it was demonstrated that embryonic tissues of the frog transplanted into “coagulable lymph” were able to develop normally. Since then, this epochal report was considered to be a milestone in biomedical research, because it demonstrated, for the first time, that the “growth of cells outside the body” was possible. In 1911, at the Rockefeller Institute for Medical Research in New York, Montrose T. Burrows and Alexis Carrel were able to grow chicken embryo cells in tissue culture. This successful experiment defined the basic protocol to standardize the in vitro culture of cells from different tissues of origin. In particular, chicken Rous sarcoma and carcinoma samples that were obtained from rats, dogs, and humans were cultured in vitro while using horse or bovine plasma [22]. In 1951, improvements in defining the biochemical requirements for the growth of physiological and transformed cells that were permitted Dr. George Otto Gey at Johns Hopkins Hospital in Baltimore to establish the first and well-known human continuous cell line, named HeLa [23]. The name “HeLa” is an acronym for Henrietta Lacks, a young black woman affected by cervical carcinoma, from which HeLa cells were derived. The establishment of the HeLa cell line can be considered as another milestone in the history of cell biology, and it opened new frontiers in the field of cancer research. Starting from their stabilization, the HeLa cells constituted the first example of “human cancer in a test tube” [24]. The establishment of the first human continuous cell line provided a standard model to study cancer pathophysiology, avoiding differences between donors and permitting the reproducibility of experimental data and the renewing of the original biological material [25]. A few years after the establishment of the HeLa cell line, in 1963, at Ibadan University in Nigeria, Robert James Valentine Pulvertaft established, from a Nigerian patient affected by Burkitt’s lymphoma, the RAJI cell line, the first human continuous hematopoietic cell line [26]. Although the RAJI cell line was successively proven to be a model system that is generated by Epstein–Barr virus infection, the definition of the culture conditions that are necessary for its growth in vitro paved the way for the stabilization of new cell lines growing in suspension. Furthermore, the availability of recombinant growth factors and conditioned media allowed, especially during the 1980s and 1990s, the stabilization of a number of hematopoietic cell lines that cover almost all steps of myeloid and lymphoid leukemia subset classification (except in cases of chronic myeloid leukemia in chronic phase) [27]. These leukemia models have made it possible to develop new therapeutic molecules that specifically target leukemic cells, similarly as in the cases of human acute promyelocytic leukemia and human chronic myeloid leukemia. In the first case, the NB4 cell line has been fundamental for the comprehension of the action of retinoic acid on the fusion gene PML-RAR alpha that was carried by malignant promyelocytic cells [28] and for the development of diagnostic assays based on the PML pattern of distribution [29]. In the second case, regarding the study of the BCR-ABL fusion protein (derived from t (9; 22)), it is important to highlight the role of the K562 cell line. This historical model has been critical in the development of STI-751 (Imatinib), the first tyrosine kinase inhibitor that is able to specifically block the catalytic site of the t (9; 22)-correlated BCR-ABL fusion protein. STI-751 has now made it possible to arrest the growth of leukemic cells, giving new expectations to many patients that are affected by this disease [30].

Overall, each new cell line has been necessary, over time, to understand step by step a new feature of cancer disease and to test the efficacy of anticancer drugs [31]. The importance of human continuous cell lines in the development of new drugs has been witnessed by the studies of the United States (US) National Cancer Institute (NCI) [32,33]. In 1986, the NCI started the human tumor cell line anticancer screening (NCI60) project to propose a novel research strategy for supplanting the use of transplantable animal tumors in anticancer drug screening [32]. The NCI60 screening service has been active since 1990 and it covers 60 different human tumor cell lines to identify and characterize the novel compounds with growth inhibition or killing activity [34]. The operation of this screen utilizes 60 different human tumor cell lines representing leukemia, melanoma, and cancers of the lung, colon, brain, ovary, breast, and prostate [35]. It is designed to screen up to 3000 small molecules (synthetic or purified natural products) per year for potential anticancer activity [36,37]. Over time, the NCI-60 project has served the global cancer research community and allowed for the identification of new important anticancer drugs, including paclitaxel, cisplatin, fludarabine, and others [38]. In conclusion, as listed in Table 1, the processes of stabilizing and characterizing novel continuous cell lines allowed for the generation of important model systems for studying cancer and developing effective drugs. Historical cell lines, such as HeLa, K562, and NB4, allowed for the publication of more than 10,000 scientific papers. Additionally, important goals for public health care have been reached, such as the generation of the anti-polio vaccine [39] and therapeutic treatments for chronic myeloid [40] and acute promyelocytic [41] leukemia. It is important to note that the role of cancer cell lines is not finished; conversely, the advent of-omics technologies and the generation of a comprehensive public database that describes the molecular and cellular alterations of each cell line at an unprecedented level renewed their importance as indispensable tools for cancer research and drug discovery, as will be discussed hereafter [42].

## 3. Cell Lines in Modern Cancer Research: Toward the “Encyclopedia” of Cell Lines

Human cancer cell lines continue to play a critical role in modern cancer research. Indeed, they are widely used as preclinical model systems for gaining mechanistic and therapeutic insight. Notably, with the advent of -omics technologies [43], recent studies have provided comprehensive databases dedicated to the characterization of most existing cell lines [44,45]. Furthermore, the online availability of the information that was derived from these studies created an important resource for the study of cancer cell lines and facilitated researchers in selecting the most appropriate in vitro model system for their research projects. In this context, it is important to consider a series of significant papers that have been published in less than 10 years.

In 2012 (and for the first time), two independent research groups that were led by Barretina et al. [14] and Garnett et al. [15] were successful in providing a large-scale genetic and pharmacological characterization of human cancer cell lines. Both of the research groups were able to perform a comprehensive characterization of several hundred cell lines using different high-throughput platforms and analytical methods. Their complimentary results confirmed that many human cell lines capture the genomic diversity of their respective cancers and, consequently, can be used as in vitro model systems of the diseases from which they were derived. In particular, in the case of Barretina et al., a large-scale genomic dataset of 947 human cancer cell lines, together with the pharmacological profiling of 24 compounds across 500 of these cell lines, was established. The resulting collection, which encompassed 36 tumor types, was termed the Cancer Cell Line Encyclopedia (CCLE) and it was made public at the website http://www.broadinstitute.org/ccle. Following this comprehensive approach, an important preliminary result that was obtained by Barretina et al. revealed the possible association between Schlafen family member 11 (SLFN11) gene expression and sensitivity to topoisomerase inhibitors. In the paper by Garnett et al., by performing a similar integration between genomic and pharmacological data, it was possible to disclose the association between the EWS-FLI1 gene translocation, which is frequently found in Ewing’s sarcoma, and sensitivity to poly (ADP-ribose) polymerase (PARP) inhibitors, which are a class of drugs currently used in clinical trials for other cancer types. Both resources that were provided by Barretina et al. [14] and Garnett et al. [15] are extremely useful when a novel defect at the DNA level or a difference in gene or protein expression is detected in a specific cancer type. Indeed, by exploring these resources, it is possible to determine whether any of the listed cell lines can be used as preclinical models to gain mechanistic and therapeutic insight, otherwise they carry no practicality in humans. The molecular profiles presented by Barretina et al. and Garnett et al. paved the way for the generation of additional resources dedicated to testing experimental hypotheses for the preclinical setup of personalized cancer medicine protocols [46]. In the following years, Iorio et al. reported how cancer-driven alterations (including somatic mutations, copy number alterations, DNA methylation, and gene expression) identified in 11,289 tumors from 29 tissues can be effectively mapped to 1001 molecularly annotated human cancer cell lines [47]. The same authors disclosed that most of the oncogenic alterations that were identified in tumor tissues are present in cancer cell lines, which confirms that they can be considered to be effective model systems for studying drug sensitivity/resistance. The genetic map defined by Iorio et al. is available as an online database through the website http://www.cancerRxgene.org.

Despite the enthusiasm generated by the aforementioned important works, it is important to consider that cell lines have important limitations, especially due to the differences in terms of gene expression, as compared to in vivo tumor tissues. Specifically, cell lines, when cultured in vitro, do not have interactions with other cell types; additionally, their growth is not under the influence of cytokines and other cell signaling molecules, and the native tissue architecture is lost. Moreover, the effects of in vivo drug distribution and metabolism are not easily matched in vitro [48]. All of these considerations indicate that sensitivity and resistance in culture might not reflect the factors that influence a drug’s action in vivo. In this context, it is also important to consider the findings of Sandberg and Ernberg [49] regarding the comparison of the NCI60 cell lines with their corresponding tumors and normal tissues. In their study, the authors demonstrated that only 34 of 60 cell lines maintained the tissue-specific upregulation of genes [49]. The authors explained their findings while considering that cell lines could be derived from a subtype of the tumor not represented in the tumor biopsy; otherwise, these cell lines have lost the differentiated phenotype of their tumor of origin or that the tumor, from which the cell line was derived, arose from a progenitor cell that lacked the gene expression that is associated with differentiated cells from that tissue. Furthermore, it cannot be excluded that the original classification might not be correct due to metastasis or cultivation problems. More recently, in 2017, Jin et al. [50] applied RNAseq technology and compared the matched tumor and cell line pairs that were derived from synovial sarcoma (SS). In their paper, the authors compared three tumor/cell line pairs from a genetically engineered mouse model of SS as well as 2 pairs from human SS tumors. The results of this comparison highlighted the considerable variation in gene expression profiles and the enrichment of microenvironment modification-related genes among those differentially expressed across all examined tumor to cell line comparisons. The findings of Sandberg and Ernberg and Jin et al. [49,50] highlight the difficulties in defining what constitutes the most appropriate preclinical model system for cancer study and drug discovery.

Klijn and colleagues [16] improved our knowledge of gene expression in cancer cell lines by performing a comprehensive transcriptional portrait while using the RNAseq approach. The authors cataloged coding and noncoding RNA expression, mutations, the expression of viral sequences, and DNA copy number changes in 675 cell lines. Notably, while using this approach, the authors determined that 1435 of 2200 fusion genes were detected for the first time and it could be further investigated while using already available cell lines. In addition, by combining gene copy number data, expression data, mutation status, and gene fusion information, the authors predicted the response to clinical compounds including MAPK/ERK kinase (MEK), phosphoinositide-3-kinase C (PI3K), and fibroblast growth factor receptor (FGFR) inhibitors in many cell lines. In this way, the authors confirmed that the data that were derived from the study of human cell lines by the application of genomic and transcriptomic technologies are critical for expediting the development of effective personalized medicine protocols [16].

In parallel to advancements in the knowledge of genetics and transcriptomics of cancer cell lines, The Cancer Genome Atlas (TCGA) database [51] revealed the great molecular diversity among tumors across and within cancer types. Therefore, understanding the functional consequence of this diversity on the treatment response has become a central task for a number of research laboratories worldwide. Consequently, it is essential to characterize the comprehensive molecular profiles of a large number of human cancer cell lines to capture the diversity that was observed in patient tumors and to elucidate the complex relationships between molecular aberrations, cancer phenotypes, and the therapeutic response [52]. In this context, using the same reverse-phase protein array (RPPA) platform that was employed for the TCGA, Jun Li et al. [17] generated a comprehensive cell line protein expression dataset for 651 independent cell lines. This study added information on protein expression, including total and post translationally modified proteins, which are arguably the most crucial molecules in the cell and, importantly, the targets of most drugs. Together with the aforementioned works that systematically characterized cancer cell lines at the DNA and RNA levels, as well as drug responses, the study by Jun Li et al. provided an additional rich resource (https://tcpaportal.org/mclp/#/) for the research community to investigate tumor behaviors in a quantitative and efficient way and to compare the differences in protein expression across cancer cell lines and to in vivo tumor tissues.

The generation of these extensive datasets highlighted the need for functional assays to identify novel targetable genes. In this sense, it is important to consider the work by Tsherniak A et al. [53] that is dedicated to the publication of results of genome-scale RNA interference (RNAi)-based loss-of-function screens (Project Achilles, https://depmap.org/portal/achilles/) to identify critical gene functions in 501 cancer cell lines. The authors identified genes whose expression is required for the proliferation or survival of subsets of these cell lines and developed an approach to identify the features that predict these gene dependencies. This cancer dependency map provides an innovative approach for defining and predicting genes that are essential for cell viability, which thereby facilitates the identification of cancer targets.

More recently, in 2019, data from two research papers added value to knowledge regarding the biology of the cancer cell lines that were included in the CCLE. The first study was published by Li et al. and focused on the metabolic diversity of 928 cancer cell lines derived from 20 cancer types [54]. The authors profiled 225 metabolites by means of liquid chromatography-mass spectrometry (LC-MS). The authors generated a resource (available at the CCLE portal, https://portals.broadinstitute.org/ccle), where unbiased association analysis can be performed by linking the cancer metabolome to genetic alterations, epigenetic features, and gene dependencies [54]. Overall, the authors proved that distinct metabolic phenotypes exist in cancer cell lines and that such phenotypes have direct implications for therapeutics targeting metabolism. The second study by Ghandi et al. expanded the characterization of the cell lines that were encompassed in the CCLE by including data on gene mutations, RNA splicing, DNA methylation, histone H3 modification, microRNA expression, and the RPPA [55]. Moreover, these data have been integrated with functional characterizations, such as drug sensitivity, short hairpin RNA knockdown, and clustered regularly interspaced short palindromic repeats (CRISPR)–Cas9 knockout data. This comprehensive approach will be extremely useful in revealing potential targets for cancer drugs and associated biomarkers.

Finally, wet laboratory researchers should have a friendly interface to explore the data on each cell line; (e.g., the Cell Model Passports (https://cellmodelpassports.sanger.ac.uk/) interface developed at the Sanger Institute (UK)) due to the complexity of the newly derived datasets [56]. This resource is a valuable tool that enables access to genomic and phenotypic datasets that were derived from cancer cell models, empowering diverse research applications. Table 2 displays an updated list of some of the existing online resources, where it is possible to have a comprehensive genetic, transcriptomic, and proteomic map for exploring most of the cell lines currently available. These resources will help researchers to determine cancer-sustaining molecular mechanisms with unprecedented depth, rigor, and speed [57]. In this way, cancer cell lines will continue to be essential for current research strategies; however, their proper use following published guidelines is mandatory, as will be discussed hereafter.

## 4. Pitfalls in the Use of Cell Lines: Cross-Contamination and Mycoplasma Infection

As discussed earlier, the exciting studies that were performed in less than 10 years corroborate the importance and usefulness of cancer cell lines in all fields of medical research, with particular reference to cancer research and drug discovery. However, it is important to highlight two outstanding problems that persist and that are related to their use: cross-contamination [19,58] and mycoplasma infection [1,59].

The first report about cross-contamination was described by Walter Nelson-Rees in 1981 [60]. In a pioneering study, he was able to prove the authenticity of each newly established cell line by means of DNA fingerprint analysis [60]. This study made it possible to show that established cell lines from different sources, such as oral cancer, intestinal adenocarcinoma, and liver carcinoma, were generated by the cross-contamination of the initial cultures with HeLa cells [25,61]. The report by Nelson-Rees was just an initial screening, but this problem continues to occur at a high rate, despite a constant stream of reports demonstrating the evidence of inter-and intraspecies cross-contamination. In a study that was conducted at Deutsche Sammlung von Mikroorganismen und Zellkulturen (DSMZ), 18% of 252 “new” continuous hematopoietic cell lines were cross-contaminated [62]. As a result of these important observations, the problem of cross-contamination became of interest in the scientific community. This quality check is extremely important for excluding the possibility of working with unwanted models and producing false data [61]. Cross-contamination may arise due to several causes, including poor technique (spread via aerosols or accidental contact), the use of unplugged pipets, sharing media and reagents among cell lines, and the use of mitotically inactivated feeder layers or conditioned medium, which may carry contaminating cells if not properly eliminated, for example, by freeze-thaw and filtration [19]. In addition, a cell line can be replaced by another as a result of a misidentification by confusing cultures during handling, mislabeling, or poor freezer inventory control. Simple errors during the labeling of culture flasks, truncation of the cell line name, or typographic errors in a published manuscript, can result in significant confusion for years after the event when another researcher attempts to use the same cell line for ongoing experimental work. The cross-contamination event may occur “early” or “late”. In the first case, the original cell line most likely never existed; in the second case, the test sample was overgrown, but other stocks of the original may still exist [63]. Unfortunately, cell lines generally become cross-contaminated early, while still within the originating laboratory [64]. This event should be correlated to the fact that, during the crisis period, cell cultures can remain in crisis for a prolonged time. During this time, if even a single cell derived from a separate and already immortalized cell line is introduced in the culture, it would rapidly take over the culture.

Cape-Davis et al. published [19] and made available online (https://en.wikipedia.org/wiki/List_of_contaminated_cell_lines) a list of 360 known cross-contaminated cell lines to address the important problem of cross-contamination. Most contaminants arise within the same species, with HeLa (as reported by Nelson-Rees approximately 40 years ago) still the most frequently encountered (29%, 106/360) among human cell lines, while interspecies contaminants account for a small, but substantial, minority of cases (9%, 33/360). In addition to the list of cross-contaminated cell lines, two important practical suggestions are provided: (i) Check the literature by searching the PubMed database while using the cell line name and “cross-contamination”; and (ii) Check the cultured cells; a cell line should be tested on arrival in a new laboratory, and all the cultures should be periodically tested while in use, before cryopreservation, and when thawed from liquid nitrogen [65]. In this context, it is important to consider that a variety of methods are available for authentication; for human cell lines, short tandem repeat (STR) profiling is the current international reference standard and it is recommended as an easy and economical way to confirm cell line identity by comparison to donor tissue or to other samples of the cell line held by laboratories worldwide [66]. An increased number of scientific journals demand that cell lines be authenticated; however, unfortunately, this practice is not universally required by all scientific journals. For this reason, it is still possible to produce incorrect results that could damage future scientific progress. More recently, the International Cell Line Authentication Committee (ICLAC, https://iclac.org/) created a website that is dedicated to the authentication of cell lines [67]. A list of and the related links to these online databases have been made available for checking the authenticity of the cell lines in terms of the STR profile, as well as the correct name and description for each known cell line (https://iclac.org/databases/). The improper use of misidentified human cells can be avoided when carrying out research projects by performing regular checks on the cell lines used, given that these resources are now available to the scientific community.

Mycoplasma contamination is the second critical issue to be discussed. This problem was first proposed in 1956, when it was discovered as a cell culture contaminant [68]. Mycoplasmas are the smallest self-replicating organisms known in terms of their morphology and genome size. They possess extremely reduced metabolic capabilities and lack a rigid cell wall. The complete lack of a cell wall is the class-defining characteristic of the Mollicutes, of which the genus mycoplasma comprises, by far, the largest group of species, and is thus commonly used as a synonym for the whole class [69]. More than 200 mycoplasma species have been described. However, merely a half dozen of these, namely, M. arginini, M. fermentans, M. hominis, M. hyorhinis, M. orale, and Acholeplasma laidlawii, account for the vast majority of all mycoplasma infections [70]. Their almost invariable obligatory dependency on eukaryotic cells and their physiological features predestine mycoplasmas to proliferate unrecognized in cell cultures for long periods of time. They gain nutrients from the rich cell culture medium and cellular metabolites, are invisible during a routine microscopic inspection of the cells, and they can pass through conventionally applied microbiological filters. They exhibit long generation times, and hence do not overgrow the eukaryotic cells. Mycoplasma infection does not usually lead to immediate cell death or to detectable turbidity of the culture medium [71]. Additionally, mycoplasmas are not affected by most of the commonly applied antibiotics that inhibit cell wall assembly or protein biosynthesis. Currently, infected cell cultures themselves are the main source of contamination. Mycoplasmas are usually transferred from an already contaminated culture to an uncontaminated culture as a result of inadequate cell culture techniques. Furthermore, laboratories with a high turnover of employees and cell lines from manifold sources are much more affected by mycoplasma contamination when compared to those with experienced personnel working with only a few cell lines from certified sources. The finding that either all or none of the cell cultures of a given laboratory are usually infected with the same mycoplasma strain also argues for a single source, from which the contaminants are spread to other cultures. Other sources of contamination (e.g., fetal bovine serum (FBS), other cell culture supplements, the incubator, and liquid nitrogen) are much less likely to contribute to the high contamination rate to a significant extent. Although the infections are usually unrecognizable during routine cell culture at the macroscopic or microscopic level, they certainly have considerable effects on the eukaryotic cells and on numerous parameters that were determined in experimental settings and are risk factors for biologically active agents that were isolated from cell cultures. As a consequence, the main measures to combat mycoplasma infections are to commit all colleagues working with cell cultures to strictly adhere to good cell culture practices [72,73], to test every incoming cell culture and all active cell cultures regularly for mycoplasma contamination (as well as for cell line cross-contamination and also optimally for viral infections), and to strictly separate the contaminated from uncontaminated cell cultures (in space and/or in time).

In conclusion, the main reasons for both cross-contamination and mycoplasma infection are the same, namely, faulty cell culture techniques, inappropriate handling of cell lines, and a lack of knowledge and information regarding the consequences and effects of contaminants. Undoubtedly, according to current guidelines, it is good practice to obtain a cell line of interest from a qualified source, such as international cell line banks [10,27,72]. These infrastructures assure a full molecular and cellular characterization, which indicate the potential fields of application of each cell line, and routine tests are applied to prevent cross-contamination as well as mycoplasma infection. In this way, these referenced cell lines assure improved research comparability, both geographically and with time. Currently, the major cell line repositories include (i) the American Type Culture Collection (ATCC) (USA); (ii) the Leibniz-Institute DSMZ; (iii) the European Collection of Authenticated Cell Cultures (ECACC); (iv) the Japanese Cancer Research Resources Bank (JCRB); the RIKEN BioResource Center (Japan); and, (v) the Korean Cell Line Bank (KCLB). In addition to these providers, we believe that institutional biobanks could play a critical role in preventing the occurrence of cross-contamination and mycoplasma infection. Indeed, the centralized management of cell lines by qualified and experienced biobank personnel could be helpful for (i) distributing cell lines received from the accredited international repositories (ATCC, DSMZ, etc.); (ii) generating a master (biobank staff only) and a working (biobank and institutional researchers) cell bank with different batches for each model system; (iii) culturing each cell line according to the provided culture conditions; (iv) routinely checking each biobanked model system for cross-contamination and mycoplasma infection; and, (v) disseminating the importance of following the published guidelines when working with cell cultures.

## 5. Selection of the Most Appropriate Model System: The Case of Hematopoietic Cell Lines

As discussed in the section dedicated to the history of cancer cell lines, over time, a number of different cell lines have been published in the scientific literature, especially in the 1970s, 1980s, and 1990s, with the aim of providing innovative in vitro model systems for experimental purposes. Recent findings highlight that the derived cell lines maintain most of the genetic alterations of the original in vivo tumor. Conversely, the transcriptomic and proteomic signatures of cell lines in most cases are completely different from their in vivo counterpart [49,50]. Subsequently, the process of selecting the most appropriate model system for studying a specific disease is complex and, according to Goodspeed et al. [13], a specific question that remains a subject of intense debate in the scientific literature is “How well do in vitro cell line models recapitulate the biologic processes of in vivo disease and drug response?” Based on the scientific literature, great effort has been taken by the research group of Dr. Hans G. Drexler at the DSMZ to address this question in the hemato-oncological field. Notably, in a series of original articles that were published during the last 25 years, the DSMZ research group facilitated the selection of the most appropriate model system for studying specific leukemia or lymphoma subtypes in the scientific community. In these articles, the authors evaluated most of the known hematopoietic cell lines with regard to their immunological, cytogenetic, molecular, and functional features to evaluate that model system was able to best represent in vitro the disease from which it was derived. In this way, hematopoietic cell lines have been evaluated for the study of chronic myeloid leukemia [27,74]; acute promyelocytic leukemia [75]; human B-cell precursor leukemia [9]; HHV-8+ primary effusion lymphomas [76]; natural killer (NK) cell leukemia-lymphoma [77]; acute leukemias with MLL gene alterations [78]; acute erythroid leukemia [79]; Waldenström’s macroglobulinemia [80]; myelodysplastic syndrome [81]; primary mediastinal B-cell lymphomas [82]; double-hit B-cell lymphomas [83]; and, Hodgkin lymphoma [84]. Approximately 637 hematopoietic cell lines have been authenticated and characterized, and this amount encompasses nearly the whole spectrum of hematopoietic cell lineages and the various stages of differentiation along the respective cellular lineage. Indeed, the currently available cell lines can be classified [85] according to their lineage, as follows: pre B-cell lines (101 model systems); B-cell lines (180 model systems); plasma cell lines (71 model systems); immature T-cell lines (59 model systems); mature T-cell lines (23 model systems); NK cell lines (11 model systems); dendritic cell lines (3 model systems); Hodgkin lymphoma cell lines (11 model systems); anaplastic large cell lymphoma (ALCL) cell lines 17 (model systems); myelocytic cell lines (77 model systems); monocytic cell lines (35 model systems); and, erythrocytic/megakaryocytic cell lines (49 model systems). Important milestones have been reached due to the correct use of hematopoietic cell lines in the hemato-oncological field, such as (i) the isolation of EBV, HIV, HTLV-I, and HHV-8 while using the RAJI, HuT78/H9, CTCL2, and BC1 cell lines, respectively; and, (ii) the cloning of chromosomal translocations and the identification of relative fusion genes t (8; 14) MYC-IGH, t (9; 22) BCR-ABL, t (2; 5) NPM-ALK, t (1; 19) E2A-PBX1, t (4; 11) MLL-AF4, inv (16) CBFB-MYH11, t (8; 21) AML1-ETO, t (15; 17) PML-RARA, and t (14; 18) IGH-BCL2 using the RAJI, K-562, SU-DHL-1, 697, RS4;11, ME-1, Kasumi-1, NB4, and DoHH2 cell lines, respectively. To date, hematopoietic cell lines continue to support the progress of scientific knowledge for the identification of the molecular and cancer alterations occurring in cases of leukemia and lymphoma, and more detailed information is available. Indeed, recently, a panel (LL-100) of 100 hematopoietic cell lines covering 22 entities of human leukemia and lymphoma, including T-cell, B-cell, and myeloid malignancies, was studied by whole-exome and mRNA sequencing [86]. This study, based on the use of unequivocally authenticated and assigned to the correct tissue leukemia-lymphoma cell lines, now provides comprehensive sequencing data that can be used to find lymphoma-subtype-characteristic copy number aberrations, mRNA isoforms, transcription factor activities, and the expression patterns of NKL homeobox genes. It is important to consider that the definition of the genomic and transcriptomic signatures of the hematopoietic cell lines is not just dedicated to their accurate classification but, more appropriately, to exploit these model systems in the postgenomic era. Indeed, when considering the continuous lowering of the costs that is needed for sequencing experiments and the very large amount of data that are provided for each disease, in vitro model systems will be necessary for defining the functional roles of the molecular alterations that were identified and helping scientists to translate this information into novel diagnostic tests or treatments.

## 6. Conclusions and Future Perspectives

**Figure 1:**
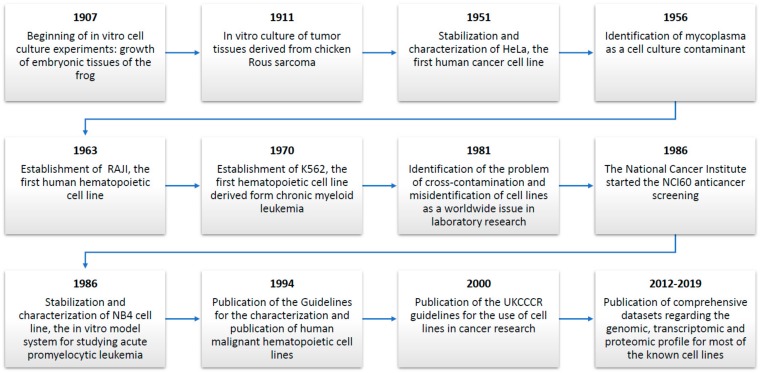
List of major breakthroughs in the historical progress of cancer cell lines.

In research laboratories worldwide, uncountable in vitro assays are performed daily while using cancer cell lines [1,2,3,25]. Figure 1 schematically represents the historical evolution of the process for the stabilization and correct use of cancer cell lines. The study of in vitro cell cultures started with fascinating historical experiments that were dedicated to the in vitro growth of amphibian and tumor tissues [21,22]. The advent of cancer cell lines, with the stabilization of the HeLa cell line, revolutionized the approach to in vitro cancer research [23,87]. The main reason for their use is their ability to provide an unlimited source of biological material that is able to grow indefinitely in vitro. Unfortunately, the stabilization of a number of cell lines, especially during the 1970s and 1980s, was followed by the generation of two pitfalls that were related to their use: cross-contamination and mycoplasma contamination [61,88]. To address these issues, which can lead to the production of false or irreproducible results, specific guidelines for the stabilization and characterization of cell lines have been published to help the scientific community [10]. Despite these known problems and the efforts of research groups and international organizations (such as the ICLAC https://iclac.org/) that were taken to help the scientific community make false or misidentified cell lines more visible and to promote awareness and authentication testing as effective ways to combat the problem, authentication and mycoplasma infection testing are not universally practiced in preclinical research.

The importance of using authenticated and mycoplasma-free cell cultures is related to the fact that cancer cell lines, under the right conditions of use, still maintain most of the cellular and molecular alterations of the original cancer [14,15,16,17]. This statement has been corroborated by recent genomic studies demonstrating a strict correlation between the genetic alterations, as evidenced by the TCGA and those present in the corresponding cell line models [17,47]. Indeed, cancer cell lines show no evidence of genetic changes in major driver mutations over long-term in vitro cultivation and embody most of the spectrum of mutations that were found in cancers, having similar patterns of chromosomal gains and losses as well as methylated regions. It is important to consider that the transcriptomic profile of cell lines greatly differs from that of the in vivo tumor, despite the general enthusiasm for the definition of the “encyclopedia” of cell line mutations and the possibility of using highly efficient genome editing technologies such as CRISPR to perform genome modifications and to make target validation and mechanistic studies much more efficient [49,50]. Most of these differences are due to the extreme differences between the in vivo microenvironment and the artificial conditions generated in vitro growing cells in suspension or in adhesion to plastic surfaces. One possible solution that needs to be tested and validated is the setup of three-dimensional (3D) matrices, where normal cells, such as stromal cells and immune cells, can be cocultured with cancer cells [89]. The optimization of culture conditions for better mimicking the in vivo environment is necessary when considering drug development in a personalized medicine context. Indeed, in the future, when the costs for DNA and RNA high-throughput sequencing will be more affordable, the treatment strategies for each patient will be established according to the molecular alterations that were identified. Consequently, the selection of the most appropriate in vitro model systems able to mimic the DNA and RNA levels of the disease will be necessary for developing new drugs or defining the role of a new diagnostic biomarker to be translated into clinical practice [10].

Prospectively, we believe that cell lines will continue to support the scientific community by helping to obtain important results that can be translated from the laboratory bench to the patient bedside and by improving public health care. As discussed in this article, the growing use of massive sequencing technologies provides a wealth of information for each disease at an unprecedented level of accuracy [55]. In this context, cancer cell lines will be important for translating the big data obtained from sequencing experiments into novel therapies or diagnostic tests for a disease. Indeed, the selection of the most appropriate in vitro model system, followed by the selection of the culture conditions to best mimic the microenvironment in vivo, will influence the quality and reproducibility of the downstream experiments that were dedicated, for example, to performing functional assays or selecting novel compounds for obtaining new drugs and therapies for diseases [31,90]. Finally, it is important to help scientists to learn more about the need to routinely check cell cultures for cross-contamination and mycoplasma infection to avoid the production of false or nonreproducible data.

## Figures and Tables

**Table 1:** Contribution of some historical cell lines to milestones in cancer research and health care. The number of publications was derived through access to PubMed (https://www.ncbi.nlm.nih.gov/pubmed/) on 28 May 2019 and applying the following search criteria: (i) (hela [Text Word]) AND cancer [Text Word]; (ii) (raji [Text Word]) AND lymphoma [Text Word]; (iii) (k562 [Text Word]) AND leukemia [Text Word]; and (iv) (nb4 [Text Word]) AND leukemia [Text Word].

Cell Line	Year of Stabilization	Number of Publications in the Cancer Field	Benefit for Public Health Care
HeLa	1953	16,843	Development of the anti-polio vaccine
RAJI	1964	1557	Definition of the mechanisms of infection by Epstein-Barr virus
K562	1976	8001	Development of treatment protocols for chronic myeloid leukemia
NB4	1991	1227	Development of treatment protocols for acute promyelocytic leukemia

**Table 2:** List of online resources with comprehensive genomic, transcriptomic, and proteomic datasets derived from cancer cell lines.

Resource Name	Website	Description	Reference
Cancer Cell Line Encyclopedia	https://portals.broadinstitute.org/ccle	The Cancer Cell Line Encyclopedia (CCLE) database was conceived to conduct a detailed genetic and pharmacologic characterization of a large panel of human cancer models (approximately 110 models). Gene expression, mutation, methylation, RNAseq and metabolomics data are downloadable.	[14]
Genomics of Drug Sensitivity in Cancer	https://www.cancerrxgene.org/	This project aims at screening >1000 genetically characterized human cancer cell lines with a wide range of anticancer therapeutics. The sensitivity patterns of the cell lines are correlated with extensive genomic data to identify genetic features that are predictive of sensitivity.	[47]
MD Anderson Cell Lines Project	https://tcpaportal.org/mclp/#/	The MD Anderson Cell Lines Project depicts the expression levels of approximately 230 key cancer-related proteins in 650 independent cell lines. This bioinformatic resource is a comprehensive resource for accessing, visualizing, and analyzing functional proteomics of cancer cell lines.	[17]
Project Achilles	https://depmap.org/portal/achilles/	Project Achilles systematically identifies and catalogs gene essentiality across hundreds of genomically characterized cancer cell lines. For each cell line, a list of genes able to alter cell survival is reported as a result of RNAi and/or CRISPR-Cas9 genetic silencing or knockout of the individual gene. Additionally, these results are linked to the genetic or molecular features of the tumors to provide a “cancer dependency map”.	[53]
Cell Model Passports	https://cellmodelpassports.sanger.ac.uk/	This resource provides large-scale genomic datasets for approximately 1200 cancer cell line and organoid models cataloged. For each model system, it is possible to display associated somatic nucleotide variants, gene expression, copy number variations or methylation data. Its accessibility format is also useful for noncomputational, wet laboratory scientists.	[56]
